# Accidental* Alcohol Poisoning Mortality in the United States, 1996–1998

**Published:** 2003

**Authors:** Young-Hee Yoon, Frederick S. Stinson, Hsiao-ye Yi, Mary C. Dufour

**Affiliations:** Young-Hee Yoon, Ph.D., and Hsiao-ye Yi, Ph.D., are senior research analysts with the Alcohol Epidemiologic Data System of the National Institute on Alcohol Abuse and Alcoholism (NIAAA), which is operated by CSR, Incorporated, Arlington, VA. Frederick S. Stinson, Ph.D., is a survey statistician in the Laboratory of Epidemiology and Biometry, Division of Intramural Clinical and Biological Research, NIAAA, Bethesda, MD. Mary C. Dufour, M.D., M.P.H., is the former deputy director of NIAAA, Bethesda, MD

**Keywords:** AOD (alcohol and other drug) poisoning, accident mortality, AODR (alcohol and other drug related) mortality, prevalence, etiology, gender differences, age differences, racial differences, educational level achieved, risk analysis, statistical data, United States

## Abstract

This study examines the prevalence and patterns of mortality resulting from unintentional poisoning by alcohol (ICD–9 code E860) in the United States. Relevant data for the most recently available years (1996 through 1998) were derived from the Multiple Cause of Death public-use computer data files compiled by the National Center for Health Statistics (NCHS). Data on deaths ascribed to alcohol poisoning as either the underlying cause or as 1 of up to 20 contributing causes were selected and analyzed. The annual average number of deaths for which alcohol poisoning was listed as an underlying cause was 317, with an age-adjusted death rate of 0.11 per 100,000 population. An average of 1,076 additional deaths included alcohol poisoning as a contributing cause, bringing the total number of deaths with any mention of alcohol poisoning to 1,393 per year (0.49 per 100,000 population). Males accounted for more than 80 percent of these deaths. The rate was lower among married than unmarried people (i.e., never married, divorced, or widowed) and was inversely related to education. Among males, the alcohol poisoning death rate was higher for Hispanics and non-Hispanic Blacks than non-Hispanic Whites. Among females, racial/ethnic differences were small, but Black women had higher alcohol poisoning death rates than White or Hispanic women. Alcohol poisoning deaths tended to be most prevalent among people ages 35 to 54; only 2 percent of alcohol poisoning decedents were younger than age 21. Among deaths with a contributing cause of alcohol poisoning, almost 90 percent had an underlying cause related to some type of poisoning from other drugs.

Beverage alcohol (i.e., ethanol) is a psychoactive drug that changes brain chemistry and can become lethal in high doses. Alcohol poisoning is an acute toxic condition resulting from exposure to excessive quantities of alcohol within a short period of time.^1^ Previous studies have suggested that the prevalence of alcohol poisoning deaths is extremely low in the United States compared with some European countries (Notzon et al. 1999; Department of Health and Human Services [DHHS] 1995; Poikolainen and Vuori 1985; Caces et al. 1991). Nevertheless, the American public has become increasingly aware of the potential public health threat of alcohol poisoning. In a recent survey by the American Medical Association (2001), more than half of the respondents (53 percent of parents and 56 percent of nonparents) expressed concern about life-threatening alcohol poisoning among college students. Despite such concern, however, the current prevalence and patterns of alcohol poisoning mortality in different population groups are not well understood.

A study by Caces and colleagues (1991) concluded that alcohol poisoning fatalities appear to be rare when such deaths are attributed to a single underlying cause.^2^ However, the number of deaths attributed to alcohol poisoning increases approximately fivefold under multiple-cause-of-death analysis, which considers all contributing causes^3^ of death in addition to the underlying cause. Because death often results from more than one cause, the multiple-cause-of-death approach has been recommended to provide a more complete analysis of mortality statistics (Van Natta et al. 1985).

The study described in this article analyzes and compares data on deaths from alcohol poisoning as either the underlying cause or a contributing cause of death in order to advance the current understanding of death from alcohol poisoning. Age-adjusted and age-specific mortality rates of alcohol poisoning deaths coded as underlying or contributing causes are presented by sex, age, race/ethnicity, marital status, and education.

## Data and Methods

This study takes data on alcohol poisoning deaths from the 1996–1998 Multiple Cause of Death public-use computer data files produced by the National Center for Health Statistics (NCHS), which uses classifying codes implemented in the *International Classification of Diseases, Ninth Revision* (ICD–9) (World Health Organization [WHO] 1978). Data from a 3-year period were used to increase the reliability of the calculations presented here. Alcohol poisoning death rates by age, sex, and race/ethnicity were calculated using population data from the U.S. Census Bureau (1999) as denominators. Denominator data for estimating alcohol poisoning death rates by marital status and educational attainment were taken from unpublished tabulations prepared by the Housing and Household Economic Statistics Division, U.S. Census Bureau, for NCHS (see Technical Notes in Peters et al. 1998; Hoyert et al. 1999; Murphy 2000).

Deaths attributed to unintentional alcohol poisoning were identified by the ICD–9 code E860, which includes seven subclassifications (E860.0 through E860.9) (WHO 1978).^4^ This study did not include deaths from intentional self-poisoning or exposure to alcohol because such deaths are not specifically coded by ICD–9.^5^

In an earlier study, Caces and colleagues (1991) included “excessive blood level of alcohol” (ICD–9 code 790.3) in their analysis. This code falls within a nonspecific category (790 codes) that documents the existence of blood alcohol without indicating specific BAC levels (NCHS 1995). The underlying causes for deaths with a contributing cause of excessive blood level of alcohol^6^ are very different from those with a contributing cause of accidental alcohol poisoning. Therefore, the code “excessive blood level of alcohol” (790.3) was excluded from the analysis described in this paper.

In most cases, the 3-year annual average of each descriptive statistic was used to simplify the result of the findings for presentation purposes. To make comparisons across sociodemographic groups, age-adjusted death rates were calculated using the 1940 standard population.^7^ Age-specific death rates provide a basis for detailed study of the variation of mortality rates across age groups.

## Results

### Alcohol Poisoning Reported as an Underlying Cause

Table 1 shows the distribution of deaths from unintentional alcohol poisoning from 1996 through 1998, classified by type of alcohol. The data show that the annual average number of deaths for which alcohol poisoning was listed as underlying cause was about 317 from 1996 through 1998. The data also indicate that slightly less than half of alcohol poisoning deaths were attributed to ethanol in alcoholic beverages (E860.0) and to “other and unspecified ethyl alcohol and its products” (E860.1). About 40 percent of alcohol poisoning deaths were caused by unspecified alcohols; the remainders were attributable largely to methyl and isopropyl alcohols. These data do not specify routes of exposure and therefore do not support any conclusions as to the contribution of alcoholic beverages to alcohol poisoning death. However, it is clear that excessive drinking was not the sole cause of alcohol poisoning deaths.^8^

### Alcohol Poisoning Reported as a Contributing Cause

Accidental alcohol poisoning was coded as a contributing factor in 3,229 deaths during 1996 through 1998 (table 2). The five most common underlying causes of death in these cases, accounting for 89 percent of the total, were classified as other types of accidental drug poisoning.^9^ The underlying cause of 39 percent of these 3,229 deaths was listed as “other drugs” (E858), a category that includes central appetite suppressants. Approximately 36 percent had an underlying cause related to accidental poisoning by “analgesics, antipyretics, or antirheumatics,” which includes heroin, methadone, and opiates (E850 codes). An additional 10 percent had underlying causes related to poisoning by various other drugs that act on the central or autonomic nervous system (E855 codes). Tranquilizers (E853 codes) and other psychotropic agents (E854 codes) accounted for a total of about 5 percent of deaths that had a contributing cause of alcohol poisoning. Deaths from alcohol-related injuries or adverse effects such as suicide, accidental drowning, motor vehicle crashes, or other accidents or injuries accounted for only about 5 percent of deaths with a contributing cause of alcohol poisoning. Thus, the relationship between alcohol-related injuries and accidental alcohol poisoning is substantially weaker than the relationship between unintentional drug poisoning and unintentional alcohol poisoning.

### Alcohol Poisoning Death Rate by Sex

Table 3 shows the number of deaths and age-adjusted death rates from alcohol poisoning for men and women from 1996 through 1998. Based on underlying cause alone, the annual average number of deaths from alcohol poisoning was 317, with an age-adjusted death rate of 0.11 per 100,000 population. In addition, alcohol poisoning was coded as a contributing cause in 1,076 deaths annually, bringing the total number of deaths with any mention of alcohol poisoning to 1,393 per year (0.49 per 100,000 population).

Among males, the annual average number of deaths from alcohol poisoning as an underlying cause of death was 252, or 80 percent of the total for both sexes. Similarly, the annual average age-adjusted death rate for males (0.18 deaths per 100,000 population) was more than four times the corresponding rate for females (0.04 deaths per 100,000 population). The annual average number of deaths in which alcohol poisoning was considered a contributing cause for males was 898, or 83 percent of the total. The annual average age-adjusted death rate from alcohol poisoning for males (0.64 deaths per 100,000 population) on a contributing-cause basis was more than five times the rate for females (0.12 deaths per 100,000 population).

### Alcohol Poisoning Death Rate by Age Group

**Figure 1:**
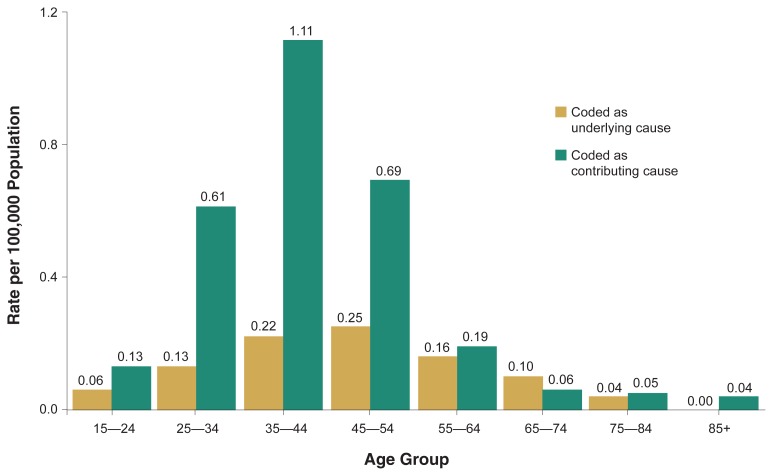
Annual average age-specific death rates of accidental alcohol poisoning (ICD–9 code: E860), United States, 1996–1998.

Figure 1 shows the annual average age-specific death rates from accidental alcohol poisoning as either the underlying cause or a contributing cause. Contrary to the popular belief that alcohol poisoning death is more prevalent among young people than older people, the death rate from this cause was highest at ages 45 to 54 (0.25 deaths per 100,000 population). The average age at death for those who died of alcohol poisoning as an underlying cause was 43.5 years.

Deaths from alcohol poisoning as a contributing cause peaked for those between ages 35 and 44 (average age at death was 39.9 years). When the age-specific rate was calculated using deaths with any mention of alcohol poisoning, the rate was highest at ages 35 to 44 (1.33 deaths per 100,000 population). From 1996 through 1998, only 2 percent of the total alcohol poisoning deaths involved people under 21 years old.

### Alcohol Poisoning Death Rate by Race/Ethnicity

**Figure 2:**
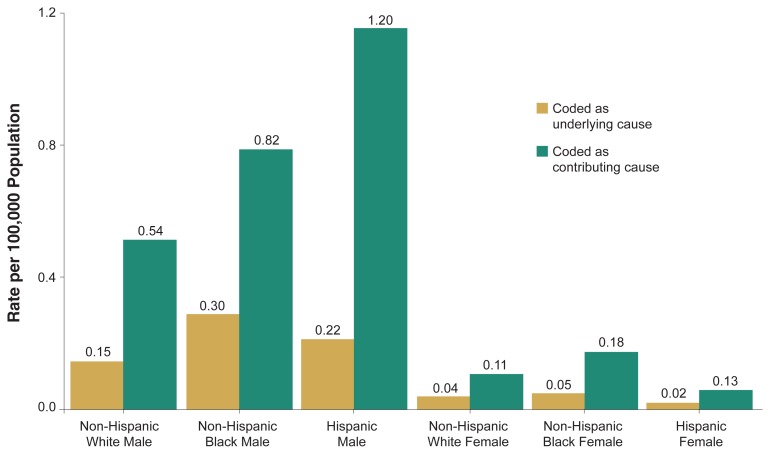
Annual average age-adjusted death rates of accidental alcohol poisoning (ICD–9 code: E860) by sex and race/ethnicity, United States, 1996–1998.

Figure 2 shows annual average age-adjusted death rates from alcohol poisoning either as the underlying cause or as a contributing cause, broken down by sex and race/ethnicity. For both underlying and contributing causes, rates of unintentional alcohol poisoning mortality among males were higher for Hispanics (0.22 and 1.20 per 100,000 population, respectively) and non-Hispanic Blacks (0.30 and 0.82 per 100,000 population, respectively) than for non-Hispanic Whites (0.15 and 0.54 per 100,000 population, respectively). In contrast, the racial/ethnic difference was much smaller among females. For both underlying and contributing causes, rates for non-Hispanic Blacks (0.05 and 0.18 per 100,000 population, respectively) were higher than those for non-Hispanic Whites (0.04 and 0.11 per 100,000 population, respectively) and Hispanics (0.02 and 0.13 per 100,000 population, respectively). Hispanic males were at the highest risk for alcohol poisoning deaths among all groups in the figure. For Hispanic males, the total combined death rate from alcohol poisoning (both underlying and contributing causes) was more than nine times as high as the combined rate for Hispanic females (1.42 per 100,000 population for males, 0.15 per 100,000 for females) and about twice as high as the combined rate for non-Hispanic White males (0.69 per 100,000 population).

### Alcohol Poisoning Death Rate by Marital Status

**Figure 3:**
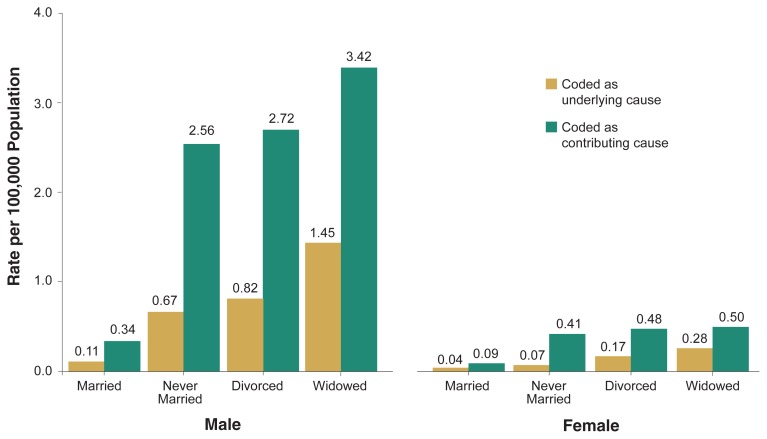
Annual average age-adjusted death rate of accidental alcohol poisoning (ICD–9 code: E860), by sex and marital status, for ages 25 years and over, United States, 1996–1998.

Figure 3 shows the annual average age-adjusted death rate from alcohol poisoning among adults ages 25 or older according to their marital status and sex. Death rates were lower for married than for unmarried people regardless of whether alcohol poisoning was the underlying or a contributing cause. For unmarried men and women, death rates were lowest among those who had never been married, higher among divorced people, and highest among the widowed.

The data also show that marital status had a stronger impact on the risk for alcohol poisoning death for males than for females. When alcohol poisoning was the underlying cause, the annual average age-adjusted death rate for widowed males (1.45 deaths per 100,000 population) was about 13 times the rate for married males (0.11 per 100,000 population), whereas the rate for the widowed females (0.28 deaths per 100,000 population) was about 7 times the rate for married females (0.04 deaths per 100,000 population). When alcohol poisoning was coded as a contributing cause, the age-adjusted death rate for widowed males (3.42 deaths per 100,000 population) was about 10 times as high as for married males (0.34 deaths per 100,000), whereas the rate for widowed females (0.5 deaths per 100,000 population) was about 6 times the rate for married females (0.09 deaths per 100,000 population).

### Alcohol Poisoning Death Rate by Education

**Figure 4:**
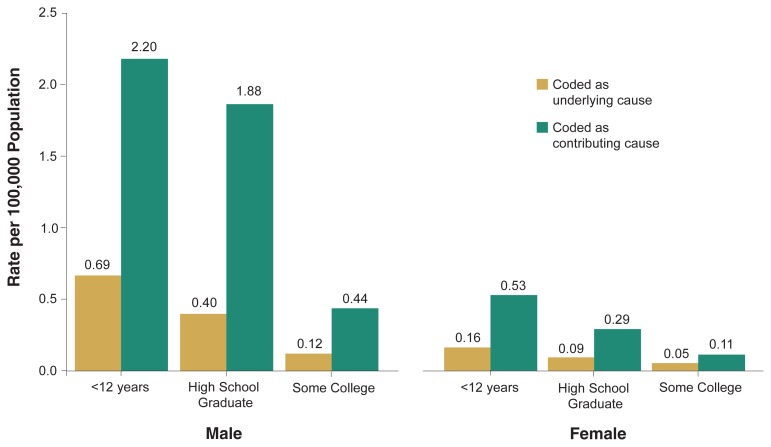
Annual average age-adjusted death rates of accidental alcohol poisoning (ICD–9 code: E860), by sex and educational attainment, among population ages 25–64 years, United States, 1996–1998.

Figure 4 shows the annual average age-adjusted death rate from accidental alcohol poisoning for adults ages 25 to 64 by sex and educational attainment. Education was inversely related to the risk of alcohol poisoning death for both men and women. In terms of underlying cause, men with less than a high school education (0.67 deaths per 100,000 population) were more than five times as likely to die from alcohol poisoning as men with at least some college education (0.12 deaths per 100,000 population). Women with less than a high school education (0.16 deaths per 100,000 population) were more than three times as likely to die from alcohol poisoning than those with at least some college education (0.05 deaths per 100,000 population). The patterns are similar when alcohol poisoning is coded as a contributing cause.

## Discussion

According to data from the Toxic Exposure Surveillance System (TESS) collected by the American Association of Poison Control Centers, 55,246 people were exposed to toxic levels of alcohol in 1998. Unintentional exposure to alcoholic beverages accounted for 5,291 (10 percent) of these cases, and intentional exposure accounted for 24,155 (44 percent) of cases (Litovitz et al. 1999).

Despite the many poisoning incidents, the number of deaths from alcohol poisoning has been low, about 317 per year from 1996 through 1998, consistent with previous research findings (Notzon et al. 1999; Poikolainen and Vuori 1985; Taylor and Hudson 1977). However, the number of deaths increases fourfold when calculated by the multiple-cause method. Among deaths with a contributing cause of alcohol poisoning, almost 90 percent had an underlying cause related to some type of poisoning from other drugs (e.g., central appetite depressants and heroin). This may result primarily from the lethal synergism between the effects of ethanol and other drugs taken concurrently (Andrews 1997; McCance-Katz et al. 1998; Ruttenber et al. 1990). However, these cases were not classified as alcohol poisoning deaths in their underlying cause because of an ICD–9 coding rule requiring that a “combination of medicinal agents with alcohol should be coded to the medicinal agent” (Lahti and Vuori 2002, p. 207). These findings underscore the importance of understanding the strong association between alcohol poisoning and other drug-related deaths. Alcohol abuse and dependence are extremely common among cocaine and heroin abusers (Brookoff et al. 1996; Carroll et al. 1993; Grant and Harford 1990; Polettini et al. 1999; Rounsaville et al. 1991; Ruttenber and Luke 1984). Furthermore, results confirm that medications such as sedatives and tranquilizers can be fatal when used in combination with alcohol (Lieber 1992; Sands et al. 1993). Based on data from official death records, Poikolainen (1984) estimated that average peak BACs at time of death were significantly lower in people who had been poisoned by a combination of ethanol and barbiturates than in people who died from ethanol poisoning alone. This suggests that even moderate drinkers may be at risk when they take certain medications and alcohol concomitantly.

The underreporting of alcohol-related deaths is well documented in the alcohol epidemiology literature (e.g., Dufour 1984; Hanzlick 1988; Hudson 1978; Nashhold and Naor 1981; Pollock et al. 1987). Hudson (1978) explores the underreporting of unintentional alcohol poisoning deaths using information about alcohol testing and acute poisoning collected for 1971 from established medical examiner systems and some of the sophisticated coroners’ offices in selected areas. He points out that, for various reasons, medical examiners tend to certify acute alcohol poisoning deaths as “natural,” which provides an opportunity for vital statistics offices to miscode these as deaths from other alcohol-related causes, such as cirrhosis, fatty liver, or other fatal complication of alcoholism. For example, the North Carolina Office of the Chief Medical Examiner office coded 45 deaths as “accidental alcohol poisoning” in 1995. The BAC level of these cases ranged from 0.26 to 0.76 grams/deciliter (personal correspondence between Young-Hee Yoon and Patricia Barnes, Office of the Chief Medical Examiner, Chapel Hill, NC, February 14, 2003). In the national vital statistics for 1995, however, only 39 deaths in North Carolina were classified as “accidental alcohol poisoning.”^10^ Without more systematic studies using national-level, post mortem forensic toxicology data, it is difficult to assess to what extent and in what manner accidental poisoning deaths are underreported in national vital statistics.

Results from this study show that middle-aged adults are at greater risk of alcohol poisoning death than younger age groups. This pattern is consistent with previous studies based on autopsy results in the United States and Finland (Taylor and Hudson 1977; Poikolainen and Vuori 1985; Lahti and Vuori 2002) showing that alcohol poisoning death is more common among experienced drinkers (i.e., alcoholics) than among inexperienced users of alcohol (e.g., youth and occasional or moderate social drinkers). Alcohol poisoning is often a complication of chronic alcoholism, and inexperienced drinkers rarely reach a lethal level of BAC (Lahti and Vuori 2002).

The results also show that among males, Hispanics and non-Hispanic Blacks have higher risk for accidental alcohol poisoning mortality than non-Hispanic Whites. This pattern is similar to the observed racial/ethnic differences in liver cirrhosis mortality (Stinson et al. 2001; Yoon et al. 2001). It is possible that drinking patterns which lead to poisoning also may contribute to liver cirrhosis mortality.

Finally, the results indicate that the risks of unintentional alcohol poisoning mortality are greater among people who are unmarried or who have completed fewer years of education. These findings are consistent with those of an earlier study conducted in Finland (Poikolainen and Vuori 1985). However, it is not clear why people who are widowed are at higher risk than those who are divorced. Further analysis is required to elucidate the various relationships between multiple demographic characteristics and alcohol poisoning death rates.

## Conclusion

Three main conclusions can be drawn from the present study. First, the data used in this study show that the number of deaths from alcohol poisoning in the United States has been low, but this low number is partly attributable to underreporting of alcohol-related deaths in general. Second, contrary to heightened public concerns about alcohol poisoning deaths among college students, characteristics associated with the highest risk of death from alcohol poisoning are: being middle-aged, unmarried, less educated, or a male from a racial or ethnic minority group. Third, given the limitation of the available data source on alcohol poisoning mortality, the present study shows a high correlation between deaths caused by alcohol poisoning and those resulting from poisoning by other drugs.

## Figures and Tables

**Table 1:** Number and Percent Distribution of Deaths for Which Accidental Alcohol Poisoning Was Coded as the Underlying Cause (ICD–9 Code: E860), United States, 1996–1998

ICD–9 Code		1996	1997	1998	3-Year Total	Annual Average	Percent Distribution of Average*
**E860 - Accidental poisoning by alcohol, not elsewhere classified**							
E860.0 - Alcoholic beverages		6	8	6	20	7	2.2
E860.1 - Other and unspecified ethyl alcohol and its products		140	164	146	450	150	47.3
E860.2 - Methyl alcohol		12	24	10	46	15	4.7
E860.3 - Isopropyl alcohol		17	17	12	46	15	4.7
E860.4 - Fusel oil		–	–	–	–	–	–
E860.8 - Other specified alcohol		2	2	1	5	2	0.6
E860.9 - Unspecified alcohol		131	127	125	383	128	40.4
	**Total**	308	342	300	950	317	100.0

* Percentages do not total 100 because of rounding.

**Table 2:** Top 10 Underlying Causes of Deaths for Which Accidental Alcohol Poisoning Was Coded as a Contributing Cause (ICD–9 Code: E860), United States, 1996–1998

Rank	ICD–9 Code	Number of Deaths 1996–1998	Yearly Average Number of Deaths 1996–1998	Percent
1	Accidental poisoning by other drugs (E858)	1,258	419	39.0
2	Accidental poisoning by analgesics, antipyretics, and antirheumatics (E850)	1,154	385	35.7
3	Accidental poisoning by other drugs acting on central and autonomic nervous system (E855)	310	103	9.6
4	Accidental poisoning by tranquilizers (E853)	76	25	2.4
5	Accidental poisoning by other psychotropic agents (E854)	70	23	2.2
6	Diseases of the circulatory system (390–459)	57	19	1.8
7	Suicide and self-inflicted injury (E950–E959)	47	16	1.5
8	Accidental drowning and submersion (E910)	45	15	1.4
9	Motor vehicle traffic and nontraffic accidents (E810–E825)	38	13	1.2
10	Other external causes of accidents, injuries, and poisoning	28	9	0.9
	Subtotal*	3,083	1,027	95.7
	Total*	3,229	1,076	100.0

* Subtotal figures are totals for the top 10 underlying causes of death for which alcohol poisoning was coded as the contributing cause of death. Total figures include all deaths in which alcohol poisoning was given as the contributing cause.

**Table 3:** Mortality from Accidental Alcohol Poisoning Coded as the Underlying or Contributing Cause (ICD–9 Code: E860), by Sex, United States, 1996–1998

	All Races, Both Sexes		All Races, Male		All Races, Female	
			
	Number of Deaths	Age-Adjusted Deaths per 100,000 Population	Number of Deaths	Age-Adjusted Deaths per 100,000 Population	Number of Deaths	Age-Adjusted Deaths per 100,000 Population
**Accidental alcohol poisoning deaths according to underlying cause**						
1998	300	0.10	245	0.17	55	0.04
1997	342	0.12	266	0.19	76	0.05
1996	308	0.11	246	0.18	62	0.04
Annual average	317	0.11	252	0.18	64	0.04
**Accidental alcohol poisoning deaths according to contributing cause**						
1998	1,158	0.40	965	0.68	193	0.13
1997	1,104	0.39	931	0.66	173	0.12
1996	967	0.34	799	0.57	168	0.12
Annual average	1,076	0.38	898	0.64	178	0.12
**Accidental alcohol poisoning deaths according to multiple cause**						
1998	1,458	0.50	1,210	0.85	248	0.17
1997	1,446	0.51	1,197	0.85	249	0.17
1996	1,275	0.45	1,045	0.75	230	0.16
Annual average	1,393	0.49	1,151	0.82	242	0.16

## Footnotes

* In the years since the 1978 publication of the *International Classification of Diseases, Ninth Revision* (ICD–9) (WHO 1978), researchers and epidemiologists have come more and more to use the word “unintentional” rather than “accidental” to describe injuries and adverse effects resulting from acts that are not deliberate. In this article the word “accidental” is sometimes used to describe unintentional alcohol poisoning when quoting or paraphrasing ICD–9 terminology.

1 Ethanol is metabolized in the liver at a rate of approximately one drink per hour, with wide variation depending on conditions. Rapid consumption of large quantities of alcohol can overload the liver’s metabolic capacity, causing the blood alcohol concentration (BAC) to rise rapidly. At BACs of 0.25 to 0.40 grams/deciliter, alcohol poisoning can lead to slurred speech, ataxia, stupor, coma, and eventually to death (Seller and Kalant 1976; Poikolainen and Vuori 1985). Alcohol poisoning is a result of extremely high levels of intoxication. With 20 drinks in 1 hour, an average male weighing 160 pounds could reach a BAC level beyond 0.40 grams/deciliter. At this point, the brain centers that control the heart and lungs are partially anesthetized and coma or death may occur (Kinney 2000). Some studies have estimated the mean lethal BAC as 0.50 grams/deciliter, but large variations in tolerance of high BAC levels have been observed. Experienced drinkers can tolerate much higher BAC levels than inexperienced drinkers. Factors such as sex, size, age, preexisting medical conditions (e.g., heart disease), whether the person vomited, and whether other drugs were used in combination with alcohol also play a role (Adinoff et al. 1988; Poikolainen 1984). When other drugs are present, even lower BAC levels may be fatal as a result of alcohol and other drug interactions (Lahti and Vuori 2002).

2 Underlying cause is defined as “the disease or injury which initiated the train of morbid events leading directly or indirectly to death, or the circumstances of the accident or violence which produced the fatal injury” (NCHS 1995, p. 12). For each death, there is only one underlying cause.

3 Contributing causes are defined as “other significant conditions contributing to death, but not resulting in the underlying cause” (NCHS 1995, p. 12). Up to 20 such conditions may be listed for each death.

4 All of the disease classification codes in this article are taken from this source.

5 With the implementation of ICD–10, alcohol poisoning codes are further classified by three categories: accidental, intentional, and undetermined intent.

6 In 1998, there were five deaths from “excessive blood level of alcohol” based on underlying cause alone. However, there were an additional 933 deaths with excessive blood level of alcohol coded as one of the contributing causes. Deaths from motor vehicle accidents (E810–E825 codes) accounted for 47 percent of these deaths; deaths from other accidents and adverse effects (E800–E809, E826–E949 codes)—accidental poisoning, drowning, and falls—accounted for 22 percent of these deaths; suicide (E950–E959 codes) accounted for 16 percent; homicide (E960–E978 codes) accounted for 4 percent; and diseases and mental disorders (001–799 codes) and other undetermined injuries (E980–E999 codes) accounted for 11 percent.

7 The 1940 standard population has been conventionally used when age-adjusting death rates prior to data year 1999.

8 The way in which a person can be exposed to various types of alcohol can vary from ingestion and dermal exposure to inhalation (Litovitz et al. 1998). Ethyl alcohol is used to produce alcoholic beverages or antifreeze in automobile radiators; methyl alcohol is used to produce such industrial products as antifreeze or solvent for gums and lacquers; isopropyl alcohol is used as a rubbing alcohol; and fusel oil is a base for perfumes and fixatives (http://www.encarta.msn.com). Because of its extreme toxicity, drinking even small amounts of methyl alcohol or inhaling or being overexposed to rubbing alcohol also can be fatal. For small children, even a mouthwash containing ethanol could be fatal (Litovitz et al. 1998, Case # 97, p. 491). Thus, unintentional alcohol poisoning encompasses a wide variety of cases, though excessive drinking appears to be the most frequent cause of death.

9 Accidental poisoning by drugs, medicinal substances, and biologicals (E850–E858) includes (a) “accidental overdose of drug, wrong drug given or taken in error, and drug taken inadvertently”; and (b) “accidents in the use of drugs and biologicals in medical and surgical procedures.” This classification excludes (a) “administration with suicidal or homicidal intent or intent to harm, or in circumstances classifiable to E980–E989” (injury undetermined whether accidentally or purposely inflicted); and (b) “correct drug properly administered in therapeutic or prophylactic dosage, as the cause of adverse effect classifiable to E930–E949” (drugs, medicinal, and biological substances causing adverse effects in therapeutic use) (see WHO 1978, p. 968).
